# Supplementary material for: Economic valuation of natural promenades in Iran using zonal travel costs method (Case study area: Gahar Lake in Lorestan Province in western Iran)
Source: PLoS One. 2020 Nov 4;15(11):e0241396. doi: 10.1371/journal.pone.0241396 (PMC7641445; doi:10.1371/journal.pone.0241396)
Supplement: S2 Data — (PDF) [file pone.0241396.s002.pdf]

Questionnaire to determine the recreational value of Gahar Lake by travel cost method

Hello

Thanking you for your cooperation, I would like to remind you that this survey is part of the studies related to my master's dissertation Ebrahim Kheiri, Master of Environmental Student, entitled " Economic Valuation of Natural Promenades in Iran Using Zonal Travel Costs Method (Case study area: Gahar Lake in Lorestan Province in Western Iran)." Therefore, please answer the questions after careful study.

Note:
This study requires respondents who have independent income
The information obtained from you is completely confidential and your cooperation in accurate and correct answers will lead to more reliable results from this research.

١) Gender: male ❐ female ❑

٢) Your age? .... years

٣) Marital status: single ☐ married ❑

٤)Number of family members?

Number of people over ١٨ years .... Number of people under ١٨ years .... people

٥) Location: Province .... City ...... City .... Region ......

٦) Education level: undergraduate ❐ diploma ❑ postgraduate ❐ bachelor ❑ master ❐ Ph.D❑

٧) If you have a university education, what is your field of study?

٨) Job status: professionals (doctors, judges, faculty)❑ employee ❐ free ❐ unemployed ❑ housewife ❐Other jobs❑

٩) Number of working hours per week .... hours

١٠) How do you usually come to the area? Single❐ Family ❐ Group and with friends❑

indifferent ❑

١١) How did you get acquainted with this area? You are a native of the area ❑ Through friends and family ❐ Through radio and television ❑ Through books, newspapers and magazines❐ Through signposts, publications and brochures❑

١٢) What is the approximate distance from your place of residence to here? ....

١٣) How long did it take you to reach the area? ....the watch

١٤) How many times have you come to this area "for a tour"?

First time ❒ ١ to ٥ times ❑ ٦ to ١٠ times ❒ More than ١٠ times ❑

١٥) if it is the first time; Would you like to come to this area again? ....

١٦) If not for the first time, how many times a year do you visit this area annually? .... per year

١٧) What were the reasons for choosing this area for recreation?

Proximity to location ❒ Ease of access ❑ Superior natural position ❒ Recommend to friends ❑

More security ❒

١٨) If there are suitable facilities for accommodation in this place, such as tents and camping, are you willing to spend the night here? Yes ☐ No

١٩) If you stay overnight, how many days will you stay in the area? .... days

٢٠) If you are temporarily staying in the area, how many hours will you stay there each time?

More than ١٢ hours ❑ ٦-١٢ hours❒ ٤-٦ hours ❑ ٢-٤ hours❒ ١-٢ hours ❑

٢١) What is the purpose of your trip and stay to this place?

Mountaineering and hiking ❑ wildlife viewing ❒ View Landscapes ❑ Plant collection❒

See natural attractions ❑ Use of open air ❒ Hunting and fishing ❑

٢٢) In what season do you most want to come to this area?

Spring❑ Summer ❒ Autumn ☐ Winter ❒

٢٣) Is it easy to access and enter the area? Yes ❒ No❑

٢٤) If the answer is negative, please state your reason?

٢٥) What means do you generally use to reach this area?

Personal device ❑ Bicycle❒ motorcycle❑ Bus ❒ Minibus ❑ Taxi❒ Car rental ❑ Walking❒

٢٦) If you came by car, how much did you spend on fuel and depreciation of your car? ....

٢٧) How often do you visit the area?

Every week❑ every month❒ every year❑ every season❒ every few years❑

٢٨) How do you assess the vegetation status of the area?

Very weak ❑ weak ❒ average ❑ good ❒ excellent ❑

٢٩) How do you evaluate the state of roads in the area?

Very weak ❑ weak ❒ average ❑ good ❒ excellent ❑

٣٠) How do you assess the security situation in the region?

Very weak ❑ weak ❒ average ❑ good ❒ excellent ❑

٣١) What is the quality of the study area in your opinion?

Very weak ❑ weak ❒ average ❑ good ❒ excellent ❑

٣٢) Were you satisfied with the area as a promenade? Yes ❒ No ❑ Not so much ❒

٣٣) What score do you give to the set of recreational, tourism and welfare facilities in this region from zero to twenty? ....

٣٤) In your opinion, what are the tourist attractions of this region?

Landscapes and landscapes ❑ Environmental values ❒ Fishing ❑ Other ❒

٣٥) In your opinion, what are the shortcomings and negative aspects of this region?

Recreational and tourism facilities ❑ Health facilities ❒ Amenities ❑ Pollution❒ Access routes ❑ Other cases❒

٣٦) the amount of monthly income:

No income❑ Less than $ ٩٥,٠٣٤ ❒ ٩٥,٠٣٤-١٩٠,٠٦٨$ ❑ ١٩٠,٠٦٨-٢٣٧,٥٨٦$ ❒ ٢٣٧,٥٨٦-٤٧٥,١٢٧ $ ❑ More than ٤٧٥.١٢٧ $ ❒

٣٧) What is your monthly expenses? .... dollars

٣٨) What percentage of your annual income do you spend on nature walks?

٣٩) How much do you spend each year to travel to this area? (Per person per household) .... dollars

٤٠) How much did it cost to get to Gahar Lake from your home?

Fuel cost .... Fare cost ....

٤١) How much do you think you will spend while you are in this area?

٤٢) Do you agree to pay an entrance fee to this area?

٤٣) If an entrance is set for this area, are you still willing to visit this area? Yes ❒ No❑

٤٤) If your answer to the previous question is yes, what is the maximum entrance fee you are willing to pay? .... Dollars

٤٥) In your opinion, what equipment and facilities should be created in this area in order to develop tourism?

٤٦) What is the maximum amount you are willing to pay annually to protect this area without any financial pressure on you now? .... Dollars

٤٧) As a visitor, what do you suggest to improve the situation in the region?

٤٨) If you can not come to Gahar Lake for fun, what other places would you choose for recreation (choose only one case)?

Amusement parks in the city ❑ Outdoor amusement parks❑ Out-of-town forest parks❐

Water recreational places such as: dams, lagoons, rivers, natural and artificial lakes, etc. ❑

Recreational-cultural places such as: museums, cinemas, cultures ... ❐

Sports places such as swimming pools, gyms and ... ❑

Other cases .... ❐

٤٩) How many times a year do you use recreational places other than Gahar Lake for recreation? ...... per year

٥٠) In the case of recreational places other than Gahar Lake, how much do you pay for each recreational use as entrance or ticket? ....Dollar

Thank you.
