# Supplementary material for: Economic valuation of natural promenades in Iran using zonal travel costs method (Case study area: Gahar Lake in Lorestan Province in western Iran)
Source: PLoS One. 2020 Nov 4;15(11):e0241396. doi: 10.1371/journal.pone.0241396 (PMC7641445; doi:10.1371/journal.pone.0241396)
Supplement: Attachment — Submitted filename: major revision.docx [file pone.0241396.s004.docx]

General comment:

This is case study of the analysis focusing Economic Valuation of Natural Promenades in Iran. The novelty of this research and research framework is unclear. To be publishable, the paper will need to be massive rewritten about following points.

216-219

This paragraph should be moved to the methods section. And this part was repeats the previous sentence 169-173. The author should rearrange and simplify.

158-159

Please indicate which 6 different education levels and which 7 income ranges were considered.

Table 1 and Figure 3 show the same thing. There is no need to keep both, one of them is enough.

253, 261-262, 266-269

For comparison purposes, please keep the same unit, the unit dollar used in introduction part.

283-284

Most visitors of Gahar lake come to the zone firstly as groups and secondly as families which can be **a proof for the high security of this regio**n. Why the larger number means much more safe? Other’s research? From your questionnaire items cannot give such conclusion.

The Results part was too simple, just list the result, just listed the results, lack of cause analysis, why such results appear? What is the point of such results.

Equation (2): VR=(-9.88-1.41TC+0.000751S+2.62I-249 3.12A+2.49E)

Why space is positive？？

in 213-214 white “the average trip cost also goes higher and the largest number of people who visit the site have less expense”. And in 285-286 white “There is a reverse relationship between the distance passed and the number of travels, so as the distance goes higher, the number of references decreases.” It is a deadly contradictions to the Equation (2) ！！

Language and presentation: The manuscript is extremely confusing to read both because of the quality of language but also because of how experimental setup and results are presented. Some sentence appears again and again.
